# Young unrelated donors confer a survival advantage for patients with myeloid malignancies compared to older siblings

**DOI:** 10.1038/s41375-025-02724-1

**Published:** 2025-08-06

**Authors:** Johannes Schetelig, Henning Baldauf, Carina Rave, Gesine Bug, Lutz P. Müller, Eva Maria Wagner-Drouet, Francis Ayuketang Ayuk, Wolfgang Bethge, Matthias Stelljes, Thomas Schroeder, Friedrich Stölzel, Edgar Jost, Christoph Schmid, Desiree Kunadt, Katja Sockel, Katharina Egger-Heidrich, Jan Moritz Middeke, Daniel Fürst, Daniel Schefzyk, Jürgen Sauter, Alexander H. Schmidt, Katharina Fleischhauer, Martin Bornhäuser, on behalf of the German Cooperative Transplant Study Group, Johannes Schetelig, Henning Baldauf, Gesine Bug, Lutz P. Müller, Eva Maria Wagner-Drouet, Francis Ayuketang Ayuk, Wolfgang Bethge, Matthias Stelljes, Thomas Schroeder, Friedrich Stölzel, Edgar Jost, Christoph Schmid, Desiree Kunadt, Katja Sockel, Katharina Egger-Heidrich, Jan Moritz Middeke, Daniel Fürst, Jürgen Sauter, Katharina Fleischhauer, Martin Bornhäuser, Deutsches Register für hämatopoetische Stammzelltransplantation und Zelltherapie (DRST), Johannes Schetelig, Gesine Bug, Lutz P. Müller, Eva Maria Wagner-Drouet, Francis Ayuketang Ayuk, Wolfgang Bethge, Matthias Stelljes, Thomas Schroeder, Friedrich Stölzel, Edgar Jost, Christoph Schmid, Daniel Fürst, Katharina Fleischhauer, Martin Bornhäuser

**Affiliations:** 1 Department of Internal Medicine I, University Hospital Carl Gustav Carus, TU Dresden, Dresden, Germany; 2 DKMS Clinical Trials Unit, DKMS Group, Dresden, Germany; 3 Goethe University Frankfurt, University Hospital, Department of Medicine 2, Frankfurt, Germany; 4 Department of Internal Medicine IV, University Hospital Halle, Halle, Germany; 5 Department of Medicine III, University Medical Center, Johannes Gutenberg University Mainz, Mainz, Germany; 6 Department of Stem Cell Transplantation, University Medical Center Hamburg-Eppendorf, Hamburg, Germany; 7 Department of Hematology, Oncology, Clinical Immunology and Rheumatology; University Hospital Tübingen, Tübingen, Germany; 8 Department of Medicine A, Hematology, Oncology, and Pneumology, University Hospital Münster, Münster, Germany; 9 University Hospital Essen, Department of Hematology and Stem Cell Transplantation, West German Cancer Center, Essen, Germany; 10 Division of Stem Cell Transplantation and Cellular Immunotherapy, University Hospital Schleswig-Holstein, Kiel, Germany; 11 Department of Hematology, Oncology, Hemostaseology and Stem Cell Transplantation, Medical Faculty, RWTH Aachen University & Center for Integrated Oncology Aachen Bonn Cologne Düsseldorf (CIO ABCD), Aachen, Germany; 12 Department of Hematology and Oncology, Augsburg University Hospital and Medical Faculty, Bavarian Cancer Research Center (BZKF) and Comprehensive Cancer Center Augsburg, Augsburg, Germany; 13 Institute for Clinical Transfusion Medicine and Immunogenetics Ulm, German Red Cross Blood Transfusion Service, Baden Wuerttemberg – Hessen and University Hospital Ulm, Ulm, Germany; 14 Institute for Experimental Cellular Therapy, University Hospital Essen, Essen, Germany

**Keywords:** Stem-cell research, Acute myeloid leukaemia, Myelodysplastic syndrome, Epidemiology

## Abstract

Donor age is one factor to optimize allogeneic hematopoietic cell transplantation (alloHCT). Therefore, we investigated whether young unrelated donors (UD) provide a benefit for older patients with myeloid malignancies compared to HLA-identical sibling donors (MSD). We performed a retrospective registry study on patients ≥50 years who received a first alloHCT between 2010 and 2020. We compared event-free survival (EFS) of patients who were transplanted from MSD aged ≥50 years versus UD aged ≤35 years who were HLA-compatible for HLA-A, -B, -C, and -DRB1. In total, we analyzed data from 3460 patients. With multivariable adjustment EFS (HR 0.86, *p* = 0.003), OS (HR 0.82, *p* < 0.001), and risk of relapse (HR 0.84, *p *= 0.018) were significantly better for HLA-compatible UD compared to MSD. No survival advantage was found, when UD with unfavorable sex or CMV constellation were compared to MSD with favorable constellations. In a meta-analysis on 9905 patients with myeloid malignancies, including ours, we found reduced risk of relapse (pooled HR 0.78, *p* = 0.006) and better EFS (pooled HR 0.89, *p* < 0.001) for young matched UD versus MSD. To select young HLA-compatible UD over older MSD may reduce relapse risk and improve survival for older patients with myeloid malignancies.

## Introduction

To select the best donor for a patient in need of an allogeneic hematopoietic cell transplantation (alloHCT) is important to maximize chances for favorable long-term results.

The first series of alloHCT for patients with leukemia were carried out by the team at the Fred Hutchinson Cancer Research Center, Seattle, Washington in the mid 70ies. HLA-identical siblings were the first donors who facilitated stable engraftment and graft-versus-leukemia (GVL) effects associated with acceptable rates of graft-versus-host disease (GVHD) translating into long-term survival [1]. Building on advances in understanding HLA and genotyping technologies, the first successful unrelated donor transplantation was performed in 1979. Since then, registries for unrelated volunteer donors were established in many countries. Today, more than 42 million donors are listed worldwide. Major advances have been made in understanding HLA-matching requirements, high-resolution HLA typing, GVHD prophylaxis and GVHD treatment. Continuous improvement of results of alloHCT was observed over the last decades, especially for HLA-compatible unrelated donor (UD) transplantation [2–4]. In 2008, we and others reported similar outcomes of HLA-compatible UD transplantation and HLA-matched sibling donor (MSD) transplantation for patients with high-risk diseases like AML [5, 6]. Yet until today, MSD have been considered first choice because of lower risk of non-relapse mortality (NRM) [7, 8].

Donor age was first described as a risk factor for transplant outcome in a retrospective CIBMTR study by Kollman et al. [9]. The impact of donor age has been confirmed in recent CIBMTR studies and donor age was incorporated into the NMDP/CIBMTR criteria for the selection of UD in 2019 [10–12]. In retrospective studies, where we evaluated the Killer cell Immunoglobulin-like Receptor (KIR) genotype of HLA-compatible UD in altogether 8,943 patients with myeloid malignancies, younger donor age was associated consistently with survival endpoints in multivariable regression modeling [13–15].

Historical evidence suggested a survival advantage of young MUD over MSD in older patients receiving alloHCT for MDS [16]. In line with this, two recently published retrospective CIBMTR studies on older patients with MDS or AML, results of transplantation with UD aged ≤35 years was compared to transplantation with MSD aged ≥50 years [17, 18]. Both studies showed a significantly reduced risk of relapse after alloHCT from younger UD. In the study on MDS patients, higher NRM after unrelated donor alloHCT prevented a survival benefit in this group [17]. In the study on AML patients, the risk of NRM changed over time [18]. Patients transplanted with young UD before 2016 showed increased NRM, whereas patients transplanted from 2016 onwards showed reduced risk of relapse compared to MSD transplantation. It is unclear how these data relate to the European practice, which differs in several ways including the frequent use of anti-thymocyte globulin as GVHD prophylaxis with unrelated donors. Here, we set out to evaluate younger UD compared to older MSD in a large cohort of older patients with myeloid malignancies in Germany.

## Methods

### Design

We conducted a retrospective cohort study on the impact of donor type for patients aged 50 years or more with myeloid malignancies registered with the Deutsches Register für Stammzelltransplantationen (DRST). The study was approved by the Ethical Committee of the TU Dresden and the Review Board of DRST. Only data from patients who had provided informed consent on the use of their medical outcome data were analyzed. The study was performed in accordance with relevant guidelines and regulations.

### Patient selection

We included data from patients with a first alloHCT for AML, MDS, MDS/MPN, or CMML performed between January 2010 and December 2020. Patients had to have an age of 50 years or more and either an UD aged 35 years or less or an HLA-identical donor aged 50 years or more. Information on HLA-A, -B, -C, and -DRB1 had to be available. Patients with cord blood transplantations and patients whose UD had mismatches at HLA-A, -B, -C, or -DRB1 were excluded.

### Definitions

For the primary efficacy analysis and for all main regression models donor type was classified as categorical variable comparing UD with an age of 35 years or lower to HLA-identical siblings aged 50 years or more. For exploratory analyses various different age cut-offs were tested. Disease risk was defined according to the Disease Risk Index (DRI) [19].

For an exploratory analysis of advantageous or disadvantageous secondary donor criteria, the following donor and recipient combinations were classified as favorable: concordant CMV status of donor and patient AND no female donor for a male patient. Patients with unfavorable secondary donor criteria were characterized either by a discordant CMV status OR a female donor for a male patient.

HLA-compatibility of unrelated donor-recipient pairs was assessed using two field information on HLA-A, -B, -C, and –DRB1. Mismatched HLA-DPB1 were classified according to the TCE group model, version 2 (https://www.ebi.ac.uk/ipd/imgt/hla/dpb.html).

Dose-intensity of the conditioning regimen was classified following Center of International Blood and Marrow Transplant Research (CIBMTR) working-group definitions [20].

### Endpoints

Event-free survival (EFS) was selected as primary endpoint. Morphological relapse, progression, or death were considered as events. Secondary endpoints were overall survival, and the cumulative incidences of relapse/progression, NRM, acute GVHD (aGVHD) grades II to IV and chronic GVHD (cGVHD) of any severity. Death without previous relapse or progression was defined as NRM. Relapse and NRM were handled as competing risks. For aGVHD and cGVHD, death and relapse/progression were defined as competing risks.

### Statistical analysis

The primary efficacy analysis was done on the impact of donor type as dichotomous variable on EFS. The primary endpoint and all secondary endpoints were evaluated in multivariable (cause-specific) Cox regression models. Additionally univariable comparisons were done with the log-rank and the Gray test. Survival probabilities were plotted as Kaplan-Meier curves. Cumulative incidences of relapse/progression, NRM, and GVHD were plotted with cumulative incidence statistics.

Multivariable models contained information on the patients’ performance status, age, disease risk index, conditioning intensity, stem cell source, T-cell depletion, and HLA mismatches. As no significant interactions (10% significance level) between these covariates and major classifiers were identified, the Cox models contained only main effects. Effects are reported as hazard ratios with 95%-confidence intervals. Point-estimates for time-to-event endpoints are reported together with 95%-confidence intervals.

The proportional hazards assumption was checked for each covariable for the main models analyzing event-free survival by means of plots of scaled Schoenfeld residuals and the test of Grambsch and Therneau [21]. Only performance status violated the PH assumption. To address this issue, we performed sensitivity analyses with a time-dependent interaction. We found marginal changes for the hazard ratios of the major classifiers and decided to present data from regression models without this interaction. The primary efficacy test was done at the 5% significance level. All additional tests were exploratory. No adjustment for multiple testing was done for those tests.

### Meta-analyses

We performed a systematic search for studies addressing the same topic referenced since first of January, 2010 in PubMed. Search terms were “donor age” AND “unrelated donors” AND (“siblings” OR “sibling donor”) AND “Hematopoietic Stem Cell Transplantation” AND (“myelodysplastic syndromes” OR “acute myeloid leukemia”) AND “retrospective studies”[O-MeSH Terms]. We retrieved 41 publications. After filtering for studies investigating large cohorts (>1000 patients) of young unrelated donors in comparison to older matched sibling donors and overlapping patient populations two publications were identified [17, 18]. We conducted meta-analyses for the main clinical endpoints EFS, OS, relapse/progression and NRM. A univariate random-effect model using the DerSimonian and Laird method was applied on the logarithmized hazard ratios [22]. The hazard ratios were derived from multivariate Cox models comparing younger 8/8 matched UD versus older MSD. The corresponding standard errors were calculated with the reported 95%-confidence intervals. The extent of heterogeneity between the studies was measured by I-squared and tested by Cochran’s Q test. The statistical significance of the pooled effect was determined by the z-test.

## Results

### Patient and donor characteristics

Medical data from 3460 patients were analyzed. The median patient age at alloHCT was 62 years ranging from 50 to 80 years. Indications for transplantation were AML or secondary AML for 61% of patients, MDS for 18% of patients, MDS/MPN for 6% of patients, and MPN for 14% of patients. Disease risk was assessed as low, intermediate, high or very high in 0.6%, 54%, 39%, and 6% of patients, respectively. In total, 2225 patients had UD and 1235 patients had MSD. HLA-DPB1 was known for 747 patients, representing 34% of the entire population of unrelated donor-recipient pairs. The majority of patients had received reduced-intensity conditioning regimens and peripheral blood stem cell products. Patient and donor characteristics are displayed in Table 1. Major differences between patients with matched UD versus MSD were, patients with UD were 2 years older at the time of alloHCT (median 63 years versus 61 years, *p* < 0.001), time between diagnosis and alloHCT was two weeks longer (median 23 weeks versus 21 weeks since diagnosis, *p* < 0.001), were more often diagnosed with secondary AML (19% versus 14%, *p* < 0.001 for the comparison over all disease categories), had received ATG as GVHD prophylaxis more often (88% versus 53%, *p* < 0.001), and had been transplanted more often in the most recent period (82% versus 76%, *p* < 0.001). The median age of UD was 27 years versus 59 years for MSD (*p* < 0.001). UD for CMV-negative patients were more often CMV-negative than MSD (35% versus 23%, *p* < 0.001 for the comparison over all CMV constellations). Also, UD were more often male than female (*p* < 0.001 for the comparison of sex constellations between UD and MSD). Information on numbers of transplantations per calendar year split by donor type are displayed in Supplemental Fig. S1.

**Table 1:** Patient characteristics.

Parameter		Total Cohort		Matched Unrelated Donors		Matched Sibling Donors		*p*-value**
		*N*	(%)	*N*	(%)	*N*	(%)	
Patient Numbers		3460	(100)	2225	(64)	1235	(36)	
Patient Sex	Male	2039	(59)	1307	(59)	732	(59)	0.93
	Female	1416	(41)	915	(41)	501	(41)	
Time from Diagnosis to HCT [weeks]	Median	22		23		21		<0.001
	IQR	15–57		16–59		14–53		
	Range	0–2463		0–2463		1–2179		
Age at HCT [years]	Median	62		63		61		<0.001
	IQR	57–67		58–68		57–65		
	Range	50–80		50–80		50–77		
Disease	AML	1518	(44)	908	(41)	610	(49)	<0.001
	sAML	599	(17)	428	(19)	171	(14)	
	MDS	631	(18)	418	(19)	213	(17)	
	MDS/MPN	84	(2)	56	(3)	28	(2)	
	CMML	130	(4)	85	(4)	45	(4)	
	MPN	498	(14)	330	(15)	168	(14)	
Disease Risk	Low	22	(0.6)	17	(0.8)	5	(0.4)	0.3
	Intermediate	1876	(54)	1189	(53)	687	(56)	
	High	1351	(39)	876	(39)	475	(38)	
	Very High	211	(6)	143	(6)	68	(6)	
Karnofsky Status	100%	782	(23)	482	(22)	300	(24)	0.06
	90%	1253	(36)	820	(37)	433	(35)	
	80%	1009	(29)	634	(28)	375	(30)	
	<80%	266	(8)	183	(8)	83	(7)	
	*Missing information*	150	(4)	106	(5)	44	(4)	
Donor Age [years]	Median	31		27		59		<0.001
	IQR	25–56		23–30		55–63		
	Range	18–79		18–35		50–79		
8/8 HLA-Match	DQB1 matched			2170	(98)			-
	DQB1 mismatched			55	(2)			
Patient-Donor Sex Constellation	Male-male	1468	(42)	1110	(50)	358	(29)	<0.001
	Male-female	563	(16)	192	(9)	371	(30)	
	Female-male	873	(25)	622	(28)	251	(20)	
	Female-female	539	(16)	289	(13)	250	(20)	
Patient-Donor CMV Serostatus	Negative-negative	1060	(31)	776	(35)	284	(23)	<0.001
	Negative-positive	318	(9)	148	(7)	170	(14)	
	Positive-negative	577	(17)	348	(16)	229	(19)	
	Positive-positive	1454	(42)	918	(41)	536	(43)	
	*Missing information*	51	(1)	35	(2)	16	(1)	
Graft Source	PBSC	3376	(98)	2174	(98)	1202	(98)	0.7
	Bone Marrow	72	(2)	43	(2)	29	(2)	
	*Missing information*	12	(0.3)	8	(0.4)	4	(0.3)	
Conditioning Intensity	Myeloablative	513	(15)	302	(14)	211	(17)	0.012
	Reduced	2790	(81)	1813	(81)	977	(79)	
	Non-myeloablative	102	(3)	68	(3)	34	(3)	
	*Missing information*	55	(2)	42	(2)	13	(1)	
T-cell depletion	no	793	(23)	239	(11)	554	(45)	<0.001
	Anti-thymocyte globulin	2602	(75)	1949	(88)	653	(53)	
	Alemtuzumab	61	(1.8)	36	(1.6)	25	(2)	
	Graft Manipulation	4	(0.1)	1	( < 0.1)	3	(0.2)	
PTCy given	Yes	51	(1.5)	30	(1.3)	21	(1.7)	0.5
	No	3409	(98.5)	2195	(98.7)	1214	(98.3)	
Year of HCT	2010–2015	687	(20)	392	(18)	295	(24)	<0.001
	2016–2020	2773	(80)	1833	(82)	940	(76)	

^*^Pearson Chi-square test of independence for categorical variables and Mann-Whitney test for continuous parameters.

*IQR* interquartile range, *AML* acute myeloid leukemia, *sAML* secondary acute myeloid leukemia, *MDS* myelodysplastic neoplasia, *MPN* myeloproliferative neoplasia, *CMML* chronic myelomonocytic leukemia, *HLA* human leukocyte antigen, *CMV* cytomegalovirus, *TBI* total body irradiation, *PBSC* peripheral blood stem cells, *HCT* hematopoietic cell transplantation, *PTCy* post-transplantation cyclophosphamide, 8/8 HLA matches refer to the loci HLA-A, -B, -C, and -DRB1.

For the whole cohort, 5-year probabilities were 43% (95% CI, 41–45%) for EFS and 49% (95% CI, 47–51%) for OS. The median follow-up was 33 months as determined with the reverse Kaplan Meier method. Altogether, 854 relapses/progression events and 1372 deaths were reported. The cumulative 5-year incidence of relapse/progression was 31% (95% CI, 29–33%) and of NRM was 26% (95% CI, 25–28%). The day 150 cumulative incidence of aGVHD grades II-IV was 31% (95% CI, 29–32%) and the 1-year incidence of cGVHD was 40% (95% CI, 38–42%).

### Impact of donor type

**Fig. 1:**
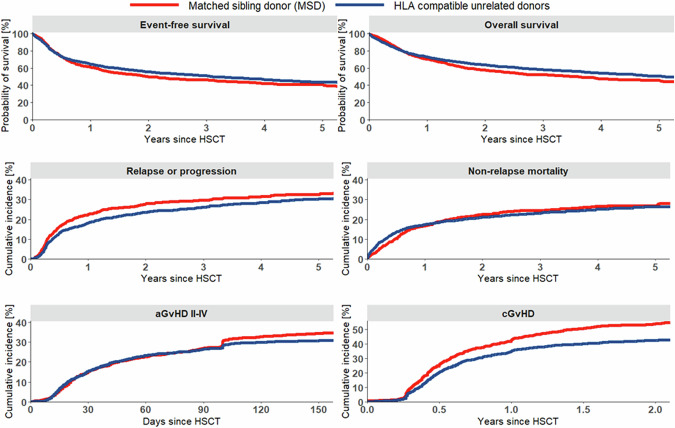
Event-free and overall survival, and cumulative incidences of relapse, non-relapse mortality, acute GVHD grades II-IV, and chronic GVHD of any severity by donor type. Kaplan-Meier curves for event-free survival and overall survival and cumulative incidence plots for relapse, non-relapse mortality, acute GVHD grades II–IV and chronic GVHD comparing patients with matched sibling donors versus HLA-compatible unrelated donors matched for HLA-A, -B, -C, -DRB1, and -DQB1.

Comparisons of EFS and OS and cumulative incidences of relapse/progression, NRM, aGVHD grades II-IV and cGVHD by donor type are shown in Fig. 1. Patients with 8/8 matched UD ≤ 35 years, including those with HLA-DQB1 mismatches, versus patients with MSD had 2-year EFS of 55% (95% CI, 53–58%) compared to 50% (95% CI, 47–53%). In line with this result, 2-year OS was 63% (95% CI, 61–66%) for patients with young UD compared to 57% (95% CI, 54–61%) for patients with MSD. Patients showed a lower risk of relapse 2-year incidence of relapse/progression of 24% (95% CI, 22–26%) after UD HCT versus 28% (95% CI, 25–31%) after MSD HCT without increased NRM. The cumulative incidences of aGVHD did not differ statistically by donor type, but patients showed lower cumulative incidences of cGVHD at one year of 35% (95% CI, 33–37%) after UD HCT versus 43% (95% CI, 40–46%) after MSD HCT. Separate results for patients with young 8/8 unrelated donors with or without HLA-DQB1 mismatches are displayed in Supplemental Fig. S2.

Per protocol, hypothesis testing was done with a multivariable Cox regression analysis for EFS. In this analysis, HCT from an UD ≤ 35 years was associated with a significant risk reduction for EFS events compared to HCT from MSD ≥ 50 years (HR 0.86, 95% CI, 0.77–0.95, *p* = 0.003). Better EFS after HCT from an UD ≤ 35 years translated into better OS (HR 0.82, 95% CI, 0.73–0.91, *p* < 0.001). Better EFS was observed in the context of reduced risk of relapse after HCT (HR 0.84, 95% CI, 0.73–0.97, *p* = 0.018) from young UD versus older MSD and of a reduced risk of cGVHD (HR 0.68, 95% CI, 0.61–0.77, *p* < 0.001). Additional risk factors with a negative impact in the multivariable regression model for EFS were higher patient age (*p* = 0.018), bone marrow as stem cell source (*p* = 0.003), Karnofsky performance status of 80% or less (*p* < 0.001) and high disease risk index (*p* < 0.001). Information on the Cox regression models for all endpoints is summarized in Table 2 and provided in detail in Supplemental Table S1.

**Table 2:** Impact of donor type on overall and event-free survival and cumulative incidences of relapse and NRM in multivariable Cox regression modeling.

Donor Type	*N*	EFS		OS		Relapse		NRM		aGVHD		cGVHD	
		HR (95% CI)	*p*	HR (95% CI)	*p*	HR (95% CI)	*p*	HR (95% CI)	*p*	HR (95% CI)	*p*	HR (95% CI)	p
Sibling	1235	1		1		1		1		1		1	
8/8 UD DQB1 matched	2170	0.86 (0.77–0.95)	0.003	0.82 (0.73–0.91)	<0.001	0.84 (0.73–0.97)	0.018	0.87 (0.75–1.01)	0.071	0.87 (0.77–0.99)	0.033	0.68 (0.61-0.77)	<0.001
8/8 UD DQB1 mismatched	55	1.26 (0.86–1.84)	.24	1.28 (0.86–1.92)	0.23	1.15 (0.67–1.96)	0.62	1.39 (0.81–2.38)	0.24	1.12 (0.71–1.78)	0.62	0.74 (0.46–1.19)	0.21
8/8 UD	2225	0.86 (0.78–0.96)	0.006	0.82 (0.74–0.92)	0.001	0.85 (0.74–0.98)	0.023	0.88 (0.76–1.02)	0.097	0.88 (0.78–1.00)	0.042	0.68 (0.61–0.77)	<0.001

^*^(Cause-specific) Cox regression models adjusted for patient age, performance status, disease risk, conditioning intensity and stem cell source.

Legend: *N* number of patients, *EFS* event-free survival, *OS* overall survival, *NRM* non-relapse mortality, *aGVHD* acute graft-versus-host disease, *cGHVD* chronic graft-versus-host disease, *HR* hazard ratio, *CI* confidence interval, *p* p-value of Wald test of Cox regression coefficient. *UD* unrelated donor; 8/8 HLA matches refer to the loci HLA-A, -B, -C, and -DRB1.

We performed additional explanatory regression analyses on subsets of patients. Information on HLA-DPB1 mismatches was available for 34% of unrelated donor-recipient pairs. In a multivariable regression model with adjustment for HLA-DPB1 mismatches, we found the biggest risk reduction for EFS (HR 0.82, 95% CI, 0.69–0.98) and OS (HR 0.72, 95% CI, 0.59–0.88) for patients with young UD with permissive HLA-DPB1 mismatches. This subset of patients showed a significantly lower risk of NRM (HR 0.74, 95% CI, 0.56–0.96, *p* = 0.026). In contrast, the small group of patients with young UD with non-permissive HLA-DPB1 mismatches showed the lowest risk of relapse (HR 0.60, 95% CI 0.38–0.93, *p* = 0.022) but no significant risk reduction in EFS, OS and NRM. Details on the HLA-DPB1 subset analysis are shown in Supplemental Table S2.

ATG-based GVHD prophylaxis was administered to 653 patients with MSD (53%) and 1949 patients with young UD (88%). In the subset of ATG-treated patients, effect estimates pointed towards risk reduction with young UD compared to MSD transplants and were in the same order of magnitude as for the entire patient population (EFS, HR 0.90, *p* = 0.1; OS HR 0.85, *p* = 0.03; risk of relapse/progression, HR 0.85, *p* = 0.08). In this subset analyses, no statistically significant risk reduction was observed for unrelated compared to related donor-recipient pairs for EFS and the risk of relapse/progression. Significant risk reductions were, however, found for OS and cGVHD as endpoints. Details on this subset analysis are shown in Supplemental Table S3.

### Head-to-head comparisons

In donor searches, sex and CMV status of the patient and the best MSD define the comparator for young UD. In order to address these settings, we evaluated three alternative settings for UD versus MSD characterized as settings where the young UD had advantageous, neutral, or disadvantageous secondary characteristics. For a young UD with a favorable sex and CMV constellation compared to an older MSD with unfavorable sex or CMV characteristics, the hazard ratio for EFS was determined as 0.84 (95% CI, 0.73–0.96, *p* = 0.01). In contrast, for a young UD with unfavorable sex or CMV characteristics compared to an older MSD with favorable sex and CMV constellation, the hazard ratio for EFS was determined as 0.93 (95% CI, 0.79–1.10, *p* = 0.38). Hazard ratios for all comparisons and including OS as endpoint are displayed in Table 3. Head-to-head comparisons based on patient and donor sex only show better EFS and OS for patients with young unrelated donors with a favorable sex constellation (see supplemental Table S4).

**Table 3:** Impact of donor type on event-free and overall survival in specific head-to-head comparisons defined by sex and CMV status constellations of donor and patient.

Sex & CMV constellation			Event-free survival		Overall survival	
Rating of Unrelated Donor	Matched Unrelated* (N)	Matched Sibling (N)	HR (95% CI)	*p*	HR (95% CI)	*p*
**Advantageous**	Favorable (1538)	Unfavorable (649)	0.84 (0.73–0.96)	0.010	0.77 (0.67–0.90)	0.001
**Neutral**	Favorable (1538)	Favorable (570)	0.79 (0.68–0.91)	0.001	0.75 (0.64–0.87)	<0.001
	Unfavorable (600)	Unfavorable (649)	1.01 (0.86–1.19)	0.92	1.01 (0.85–1.20)	0.95
**Disadvantageous**	Unfavorable (600)	Favorable (570)	0.93 (0.79–1.10)	0.38	0.96 (0.81–1.15)	0.68

Favorable and unfavorable sex- and cytomegalovirus (CMV) serostatus-constellations of donor and patient were defined as follows: *favorable* was defined as concordant CMV status of donor and patient AND no female donor for a male patient; unfavorable was defined by a discordant CMV status OR a female donor for a male patient. Numbers (N) of transplantations available for the comparisons are given in brackets. HLA-compatible unrelated donors were matched for HLA-A, -B, -C, -DRB1, and DQB1. The hazard ratios for the donor type comparisons (reference category: HLA-identical sibling donor) are taken from multivariable Cox regression models adjusted for patient age, performance status, disease risk, conditioning intensity and stem cell source.

**Fig. 2:**
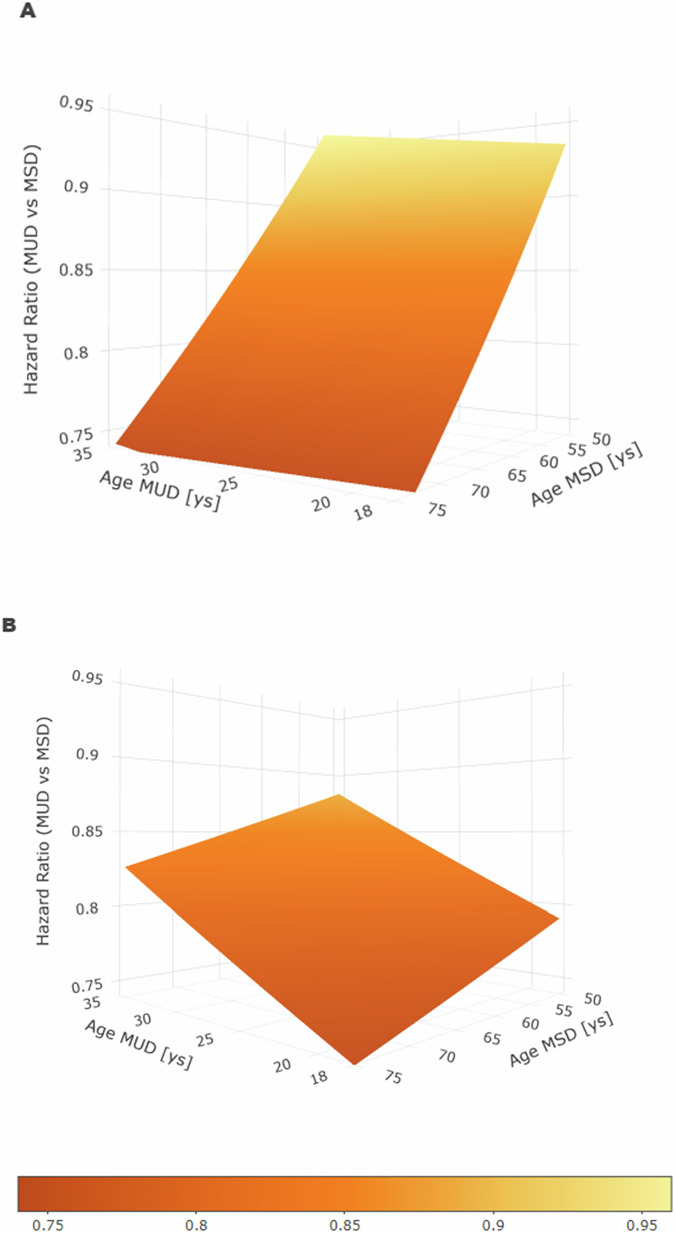
Impact of donor age as continuous variable for event-free and overall survival. Three-dimensional plot of hazard ratio for HLA-compatible unrelated donor versus matched sibling donor depending on the age of both donors. HLA-match was defined as compatibility for HLA-A, -B, -C, -DRB1 and -DQB1. Age of matched unrelated donors (MUD) is plotted on the x-axis and age of matched sibling donors (MSD) on the z-axis. The y-axis gives the resulting hazard ratio for event-free survival (Panel **A**) and overall survival (Panel **B**). The color code corresponds to the segment of the y-axis. Hazard ratios are taken from multivariable Cox regressions models for event-free survival and overall survival modeled with linear effects for unrelated donor and sibling donor age as additional continuous variables.

To better assess the impact of age, we modeled donor age as a continuous variable. The resulting hazard ratio of a given age difference between a younger UD and an older MSD is shown in Fig. 2. In this model, an age difference of 57 years (18 years old UD compared to 75 years old MSD) was associated with a 28% risk reduction for EFS (Fig. 2A) and a 26% risk reduction for OS (Fig. 2B). The smallest age difference in this study of 15 years (35 years old UD compared to 50 years old MSD) was associated with a 4% risk reduction with respect to EFS and a 12% risk reduction for OS.

### Meta-analyses

**Fig. 3:**
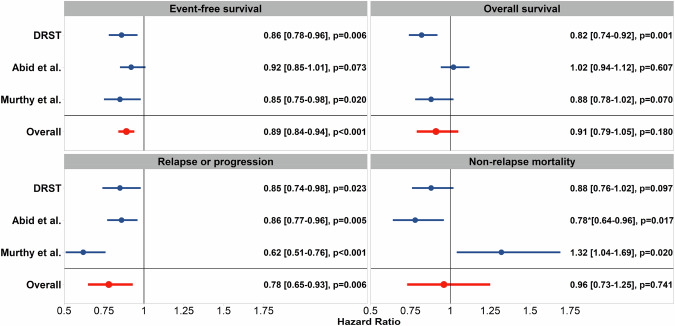
Meta-analyses on large registry studies comparing impact of young unrelated donors to older sibling donors for patients with myeloid malignancies on event-free and overall survival, risk of relapse and of non-relapse-mortality. Forest-plot for the meta-analyses on large contemporary registry studies on the impact of donor type and age on transplant outcome for patients with myeloid malignancies. The hazard ratios of the individual studies and pooled hazard ratios are shown for the comparison of matched unrelated donor vs. matched sibling donor transplantation for event-free survival, overall survival, relapse and non-relapse mortality. The hazard ratios are shown together with 95% confidence intervals. The *p*-value reflects the statistical significance of the pooled hazard ratio derived from a random-effect meta-analysis. * In the study of Abid et al. Non-Relapse Mortality was reported for two time periods, 2011 to 2015 and 2016 to 2018 (18). This meta-analysis used the result of the more recent period.

We identified two registry trials which met the selection criteria [17, 18]. Altogether, data on 9905 patients with alloHCT performed since 2010 for AML or MDS, including the data from this study were pooled for meta-analyses. Figure 3 illustrates the hazard ratios of the individual studies and the pooled hazard ratios for each endpoint. For EFS, a statistically significant benefit of younger 8/8 matched UD compared to older MSD was found, with a pooled HR of 0.89 (95% CI, 0.84–0.94, *p* < 0.001). No evidence of heterogeneity was observed (I-squared 0%, *p* = 0.52). A significant reduction in risk of relapse/progression was observed across the three studies including ours for young matched UDs, with a pooled HR of 0.78 (95% CI, 0.65–0.93, *p* = 0.006). The meta-analyses also indicated that young matched UD were associated with a reduced risk of mortality, although this advantage was not statistically significant (pooled HR for OS, 0.91, 95% CI, 0.79–1.05, *p* = 0.18). The pooled HR for NRM was 0.96 (95% CI, 0.73–1.25, *p* = 0.74). I-squared values of 80% (*p* = 0.007) for OS, 76% (*p* = 0.013) for relapse/progression and 82% (*p* = 0.004) for NRM indicated significant heterogeneity between the three studies. Major differences were higher disease risk, poorer performance score, and more use of reduced-intensity conditioning and ATG for GVHD prophylaxis in our cohort compared to both CIBMTR cohorts (see also supplemental Table S5).

## Discussion

This large retrospective study shows improved survival and a lower risk of relapse for patients with myeloid malignancies transplanted in Germany with young matched UD compared to older MSD. The risk reduction was 14% for EFS (*p* = 0.003), 16% for relapse/progression (*p* = 0.018), and 18% for OS (*p* < 0.001). Notably, this is the first large retrospective multicentre study, which shows improved EFS and OS with young UD resulting from a reduced risk of relapse and nominally lower NRM (HR 0.87, *p* = 0.071). The lower risk of relapse after UD alloHCT for patients with myeloid malignancies is in line with data from two independent CIBMTR studies [17, 23]. To integrate findings of our study with published data, we performed a systematic literature search and identified two contemporary studies addressing similar research questions [17, 18]. Data on altogether 9905 patients with myeloid malignancies, including data from this study, were combined for meta-analyses. Pooled hazard ratios showed consistently an 11% risk reduction for EFS (*p* < 0.001) and a 22% reduction for the risk of relapse/progression (*p* = 0.006). I-square values for the endpoints OS and NRM indicated heterogeneity between the three cohorts from Europe and the US, possibly related to differences in patient selection and choices for conditioning and GVHD prevention, in particular the frequent use of ATG (see Supplemental Table S5). Whereas contemporary studies from Canada and the US did not show improved survival with HLA-compatible young UD over older MSD, still comparable outcomes with either donor type were reported [24, 25]. Donor age also impacted on outcome with PTCy-based GVHD prophylaxis as reported in one EBMT registry study [26]. And Ramdial et al. reported improved survival, and lower risk of relapse and NRM with young HLA-matched UD versus older MSD in the context of PTCy-based GVHD prophylaxis using propensity score matching compared to older MSD in a small single center cohort [27].

We sought to further understand the relative contribution of donor age, donor sex, and CMV status. If multiple young UD are available for a given patient, a donor with a favorable sex or CMV constellation may be selected. This is rarely possible among HLA-identical siblings. The greater possibility for choice with UD explains higher percentages of favorable sex- and CMV-constellations in UD compared to MSD transplantation. This was also observed in our study. Yet for a given patient, the choice has to be made between the best actual matched sibling and the best actual unrelated donor. To address this challenge, we grouped donors according to sex and CMV constellations and performed head-to-head comparisons between favorable or unfavorable related and UD (see Table 3 and supplemental Table S4). Our results suggest that whenever the sex and CMV status is advantageous for an HLA-compatible UD, a survival advantage can be assumed. With disadvantageous sex or CMV-status of a young UD survival outcomes are on par with older MSD.

Studies from the early 2000s did not find advantages for younger UD over MSD. Alousi et al. published a comparison on alloHCT performed between 1995 and 2005 without selecting for large age differences between unrelated and related donors and did not observe a survival advantage [7]. Kumar et al. compared the outcome of alloHCT between 2000 and 2012 with young male UD (median age 32 years, range, 18 to 61 years) versus HLA identical sisters with a history of pregnancy (median age 48 years, range, 3 to 82 years). Also, this comparison did not show a survival advantage for male UD [8]. Both studies did not compare very young UD (defined as having an age of 35 years or less) to MSD of 50 years or more. With the selection criteria of our study (and of recently published CIBMTR cohorts) the minimum possible age difference was 15 years. In our cohort, UD were in median 22 years younger than the MSD. This design gave UD a greater competitive advantage but also mirrors current practice characterized by selecting the youngest available unrelated donor. Notably, a recent study from Japan on alloHCT for AML performed between 2013 and 2021 with 5704 patients over 50 years of age showed a reduced risk of relapse and increased leukemia-free survival for patients with HLA-A, -B, -C, and -DRB1 compatible unrelated donors when compared to patients with MSD regardless of an age difference [28].

If HLA-matched, siblings share identical haplotypes of the major histocompatibility complex (MHC) located on chromosome 6. In contrast, matched UD usually differ with respect to several classical and non-classical HLA genes not used for matching. In addition, UD show more differences for minor histocompatibility antigens than matched siblings [29]. Greater immunogenic differences between donor and patient result in a higher risk for GVHD but may also translate into stronger GVL effects. Results of this study suggest that those mismatches may be beneficial by lowering the risk of relapse when potent strategies for GVHD-prophylaxis are used. It is unclear, if more donor lymphocyte infusions in the related donor setting could compensate for this, especially, in the light of higher incidence of chronic GVHD among patients with related donors observed in this study. Whether adoptive immunotherapy could be harnessed to decrease the risk of relapse after matched sibling donor transplantation should be further investigated.

Superior fitness of hematopoietic stem cells from younger individuals to reconstitute hematopoiesis was demonstrated already decades ago [30]. Nevertheless, age-related changes of the lympho-hematopoietic system are multifaceted. Changes may affect stem and progenitor cells, immune effector cells, regulatory T-cells and further factors defining immune responsiveness. Mechanistically, it is unclear which aging-related cellular or subcellular changes are responsible for the advantage of younger donor cells. Yet, it has been demonstrated elegantly, that transplantation of hematopoietic stem cells from their native environment to another individual exaggerates selective pressure and accelerates loss of clonal diversity – stress which younger cells might better endure [31].

This study has several limitations. GVHD-prophylaxis differed between the two donor types. However, in a subset analysis in patients who all had received ATG, we observed the same overall pattern of results (Supplemental Table S3). Still, it is possible that e.g. post-transplant management differed between the two donor groups (e.g. taper of immunosuppression). Owing to the limitations of retrospective analyses, associations should not be interpreted as causal effects and thus, we cannot exclude that differences in patient management accounted for the survival advantage of UD and not immunogenic differences. This study was performed on patients treated in Germany, who are almost exclusively non-Hispanic white. Chances for identifying multiple HLA-matched UD for other ethnicities are smaller [32]. Next, letermovir was approved for CMV prophylaxis after alloHCT for CMV seropositive patients by the European Medicines Agency in 2018. Up to now, it is unclear, if letermovir prophylaxis changed the impact of donor CMV status as a risk factor for patient outcome. We were not able to address this question. Further, we had no data on measurable residual disease (MRD) in patients with AML transplanted in complete remission, which is known as a negative risk factor reflecting adverse AML biology. Consequentially, our effect estimates are not adjusted for this factor. Finally, numbers of patients with PTCy as GVHD prophylaxis were too small in our dataset to allow meaningful analysis of its impact. PTCy may change the weight of immunogenic risk factors, e.g. of non-permissive DPB1 mismatches [33]. Consequentially, more studies which aim at the ranking of donor selection criteria in the context of PTCy-based GVHD prophylaxis are warranted.

In conclusion, selecting young HLA-compatible unrelated donors over older matched sibling donors may improve survival chances after alloHCT for older patients with myeloid malignancies. This statement is based on results from three large independent studies which analyzed data on 9905 patients. Subgroup analyses from our study showed that the survival advantage was offset, when unrelated donors with unfavorable sex or CMV constellation were compared to sibling donors with favorable constellations. In this subset, results were comparable and additional criteria such as donor accessibility, cost, and safety of older stem cell donors should be weighed. In contrast, in settings where an unrelated donor aged 35 years or less compares advantageous or neutral with respect to sex and CMV status to an HLA-identical sibling donor aged 50 years or more, the young unrelated donor should be preferred. This finding argues in favor of a change in clinical practice, where MSD regardless of age were preferred over unrelated donors. In addition, donor safety may argue in individual cases in favor of asking a young unrelated volunteer instead of an older sibling for stem cell donation. Further research is warranted to better understand the underlying biological mechanisms, e.g. differences in the kinetics of immune reconstitution, and to study the impact of donor type in other diseases.

## Supplementary information

Supplement

## Data Availability

Data may be accessed via the corresponding author by academic research groups beginning 12 months and ending 48 months following article publication. Access is conditional on approval by the data access committee of the DRST.
