# Supplementary material for: Young unrelated donors confer a survival advantage for patients with myeloid malignancies compared to older siblings
Source: Leukemia. 2025 Aug 6;39(10):2523–32. doi: 10.1038/s41375-025-02724-1 (PMC12463653; doi:10.1038/s41375-025-02724-1)

Supplement to

**Young unrelated donors confer a survival advantage for patients with myeloid malignancies compared to older siblings**

This appendix has been compiled to give readers further insights into a retrospective registry study and meta-analysis comparing outcomes after alloHCT for older patients with myeloid malignancies who received hematopoietic stem cells from either an HLA-matched unrelated donor or a matched sibling donor.

Johannes Schetelig, Henning Baldauf, Carina Rave, Gesine Bug, Lutz P. Müller, Eva Maria Wagner-Drouet, Francis Ayuketang Ayuk, Wolfgang Bethge, Matthias Stelljes, Thomas Schroeder, Friedrich Stölzel, Edgar Jost, Christoph Schmid, Desiree Kunadt, Katja Sockel, Katharina Egger-Heidrich, Jan Moritz Middeke, Daniel Fürst, Daniel Schefzyk, Jürgen Sauter, Alexander H. Schmidt, Katharina Fleischhauer, Martin Bornhäuser on behalf of the German Cooperative Transplant Study Group and the Deutsches Register für hämatopoetische Stammzelltransplantation und Zelltherapie (DRST)

Table of contents

[Table S1. Multivariable Cox model for 8/8 matched young unrelated donor transplantation versus matched sibling donor transplantation on overall and event-free survival and cumulative incidences of relapse and NRM 3](#_Toc202080125)

[Table S2. Multivariable Cox model for 8/8 matched young unrelated donor transplantation versus matched sibling donor transplantation adjusted for DPB1 mismatches 4](#_Toc202080126)

[Table S3. Multivariable Cox model for 8/8 matched young unrelated donor transplantation versus matched sibling donor transplantation in subset of patients with ATG-based GVHD prophylaxis 5](#_Toc202080127)

[Table S4. Impact of donor type on event-free and overall survival in specific head-to-head comparisons defined by the sex constellation of donor and patient 6](#_Toc202080128)

[Table S5. Meta-analyses on large registry studies comparing impact of young unrelated donors to older sibling donors for patients with hematologic malignancies 7](#_Toc202080129)

[Figure S1. Numbers of HLA-identical sibling and HLA-compatible unrelated donor transplants over time. 8](#_Toc202080130)

[Figure S2. Event-free and overall survival, and cumulative incidences of relapse, non-relapse mortality, acute GVHD grades II-IV, and chronic GVHD of any severity by donor type 9](#_Toc202080131)

## Table S1. Multivariable Cox model for 8/8 matched young unrelated donor transplantation versus matched sibling donor transplantation on overall and event-free survival and cumulative incidences of relapse and NRM

| **Covariable** | **Level** | **EFS** | | **OS** | | **Relapse** | | **NRM** | |
| --- | --- | --- | --- | --- | --- | --- | --- | --- | --- |
|  |  | HR  (95% CI) | p | HR  (95% CI) | p | HR  (95% CI) | p | HR  (95% CI) | p |
| Donor type | MSD | 1 |  | 1 |  | 1 |  | 1 |  |
|  | 8/8 UD | 0.86  (0.77-0.95) | .003 | 0.82  (0.73-0.91) | <0.001 | 0.84  (0.73-0.97) | .018 | 0.87  (0.75-1.01) | .07 |
| DQB1-Mismatch | 0 | 1 |  | 1 |  | 1 |  | 1 |  |
|  | 1 | 1.47  (1.01-2.14) | .045 | 1.57  (1.06-2.34) | .026 | 1.36  (0.80-2.31) | .26 | 1.60  (0.94-2.72) | .09 |
| Patient age | per 10 year increase | 1.10  (1.02-1.20) | .018 | 1.18  (1.08-1.29) | <0.001 | 0.98  (0.87-1.09) | .68 | 1.27  (1.12-1.43) | <0.001 |
| Stem cell source | PBSC | 1 |  | 1 |  | 1 |  | 1 |  |
|  | BM | 1.57  (1.16-2.11) | .003 | 1.46  (1.07-2.01) | .019 | 1.86  (1.26-2.73) | .002 | 1.27  (0.79-2.03) | .32 |
|  | missing | 2.13  (1.10-4.10) | .024 | 1.22  (0.51-2.93) | .66 | 2.67  (1.19-5.98) | .017 | 1.49  (0.48-4.65) | .49 |
| Performance status | 100 | 1 |  | 1 |  | 1 |  | 1 |  |
|  | 80-90 | 1.17  (1.03-1.33) | .015 | 1.28  (1.11-1.47) | .001 | 0.97  (0.83-1.15) | .75 | 1.51  (1.23-1.85) | <0.001 |
|  | <80 | 1.70  (1.39-2.07) | <0.001 | 2.12  (1.72-2.62) | <0.001 | 0.99  (0.74-1.34) | .97 | 2.94  (2.23-3.87) | <0.001 |
|  | missing | 1.50  (1.17-1.94) | .002 | 1.62  (1.23-2.13) | .001 | 1.02  (0.71-1.48) | .90 | 2.35  (1.64-3.36) | <0.001 |
| Conditioning | RIC | 1 |  | 1 |  | 1 |  | 1 |  |
|  | MAC | 0.89  (0.77-1.03) | .12 | 0.91  (0.77-1.06) | .24 | 0.85  (0.70-1.04) | .12 | 0.94  (0.76-1.16) | .55 |
|  | NMA | 1.30  (0.97-1.74) | .08 | 1.22  (0.88-1.69) | .23 | 1.66  (1.13-2.43) | .010 | 0.97  (0.61-1.54) | .91 |
|  | missing | 0.83  (0.55-1.24) | .37 | 0.79  (0.51-1.24) | .31 | 0.96  (0.56-1.63) | .87 | 0.70  (0.37-1.31) | .26 |
| Disease risk index | intermediate | 1 |  | 1 |  | 1 |  | 1 |  |
|  | low | 0.65  (0.27-1.58) | .35 | 0.50  (0.16-1.55) | .23 | 0.97  (0.36-2.61) | .95 | 0.29  (0.04-2.05) | .21 |
|  | high | 1.67  (1.50-1.85) | <0.001 | 1.61  (1.44-1.81) | <0.001 | 1.90  (1.65-2.20) | <0.001 | 1.43  (1.23-1.67) | <0.001 |
|  | very high | 3.13  (2.63-3.73) | <0.001 | 3.24  (2.70-3.89) | <0.001 | 4.04  (3.19-5.11) | <0.001 | 2.34  (1.80-3.05) | <0.001 |

Abbreviations: N, number of donors; EFS, event-free survival; OS, overall survival; NRM, non-relapse mortality; HR, hazard ratio; MSD, matched sibling donor; UD, unrelated donor; PBSC, peripheral blood stem cells; HCT, hematopoietic cell transplantation; BM, bone marrow; RIC, reduced intensity conditioning; MAC, myeloablative conditioning; NMA, non-myeloablative conditioning;

## Table S2. Multivariable Cox model for 8/8 matched young unrelated donor transplantation versus matched sibling donor transplantation adjusted for DPB1 mismatches

| **Donor Type** | **N** | **EFS** | | **OS** | | **Relapse** | | **NRM** | |
| --- | --- | --- | --- | --- | --- | --- | --- | --- | --- |
|  |  | HR  (95% CI) | p | HR  (95% CI) | p | HR  (95% CI) | p | HR  (95% CI) | p |
| Sibling | 1235 | 1 |  | 1 |  | 1 |  | 1 |  |
| 8/8 UD with compatible DPB1 | 226 | 0.88  (0.71-1.08) | .22 | 0.85  (0.68-1.07) | .18 | 0.87  (0.65-1.17) | .37 | 0.87  (0.64-1.19) | .39 |
| 8/8 UD with permissive DPB1 | 385 | 0.82  (0.69-0.98) | .031 | 0.72  (0.59-0.88) | .001 | 0.90  (0.71-1.14) | .40 | 0.74  (0.56-0.96) | .026 |
| 8/8 UD with non- permissive DPB1 | 136 | 0.80  (0.61-1.06) | .12 | 0.85  (0.64-1.15) | .30 | 0.60  (0.38-0.93) | .022 | 1.01  (0.71-1.45) | .94 |
| 8/8 UD with unknown DPB1 | 1478 | 0.87  (0.77-0.97) | .011 | 0.83  (0.73-0.93) | .002 | 0.84  (0.73-0.98) | .030 | 0.89  (0.75-1.05) | .15 |

Legend: N, number of donors; EFS, event-free survival; OS, overall survival; NRM, non-relapse mortality; HR, hazard ratio; UD, unrelated donor;

## Table S3. Multivariable Cox model for 8/8 matched young unrelated donor transplantation versus matched sibling donor transplantation in subset of patients with ATG-based GVHD prophylaxis

| **Donor Type** | **N** | **EFS** | | **OS** | | **Relapse** | | **NRM** | |
| --- | --- | --- | --- | --- | --- | --- | --- | --- | --- |
|  |  | HR  (95% CI) | p | HR  (95% CI) | p | HR  (95% CI) | p | HR  (95% CI) | p |
| Sibling | 649 | 1 |  | 1 |  | 1 |  | 1 |  |
| 8/8 UD & HLA-DQB1-Match | 1889 | 0.90  (0.79-1.03) | .12 | 0.85  (0.74-0.99) | .033 | 0.85  (0.71-1.02) | .08 | 0.96  (0.78-1.18) | .69 |
| 8/8 UD & HLA-DQB1-MM | 50 | 1.37  (0.92-2.05) | .12 | 1.46  (0.96-2.23) | .08 | 1.09  (0.61-1.97) | .77 | 1.75  (1.01-3.05) | .047 |

Legend: N, number of donors; EFS, event-free survival; OS, overall survival; NRM, non-relapse mortality; HR, hazard ratio; UD, unrelated donor; HLA, human leukocyte antigen; MM, mismatch

## Table S4. Impact of donor type on event-free and overall survival in specific head-to-head comparisons defined by the sex constellation of donor and patient

| **Sex constellation** | | | **Event-free survival** | | **Overall survival** | |
| --- | --- | --- | --- | --- | --- | --- |
| **Rating of Unrelated Donor** | **10/10 Matched Unrelated (N)** | **Matched  Sibling (N)** | HR (95% CI) | p | HR (95% CI) | p |
| **Advantageous** | Favorable (1973) | Unfavorable (371) | 0.81 (0.69-0.95) | 0.011 | 0.75 (0.63-0.89) | 0.001 |
| **Neutral** | Favorable (1973) | Favorable (859) | 0.88 (0.77-0.99) | 0.033 | 0.85 (0.74-0.97) | 0.013 |
|  | Unfavorable (187) | Unfavorable (371) | 0.93 (0.72-1.20) | 0.59 | 0.89 (0.68-1.17) | 0.40 |
| **Disadvantageous** | Unfavorable (187) | Favorable (859) | 0.97 (0.77-1.22) | 0.81 | 0.96 (0.74-1.23) | 0.73 |

For the comparison of specific sex-constellations of donor and patient the following definitions applied: *favorable* was defined as concordant sex or male donor for a female patient; *unfavorable* was defined as female donor for a male patient. Numbers (N) of transplantations available for the comparisons are given in brackets. The hazard ratios for the donor type comparisons (reference category: HLA-identical sibling donor) are taken from multivariable Cox regression models adjusted for patient age, cytomegalovirus (CMV) constellation of patient and donor, performance status, disease risk, conditioning intensity, and stem cell source.

## Table S5. Meta-analyses on large registry studies comparing impact of young unrelated donors to older sibling donors for patients with hematologic malignancies

| **Characteristic** | | **DRST** | | **Murthy et al.^16^** | | **Abid et al.^17^** | |
| --- | --- | --- | --- | --- | --- | --- | --- |
|  |  | MSD | 8/8 mUD | MSD | 8/8 mUD | MSD | 8/8 mUD |
| Cohort/Registry | | DRST (Germany) | | CIBMTR | | CIBMTR | |
| N | | 1235 | 2225 | 646 | 1115 | 1736 | 2948 |
| Year of HCT | range | 2010-2020 | | 2011-2017 | | 2011-2018 | |
| Follow-up time [ys] | median [range] | 2.9 [0-11.7] | 2.8 [0-11.3] | 4 [0.3-8.3] | 3.8 [0.3-8.2] | 4.3 [0.5-9.4] | 4.2 [0.3-9.4] |
| Patient age [ys] | median [range] | 61 [50-76] | 63 [50-79] | 65 [50-77] | 67 [50-80] | 61 [50-80] | 63 [50-84] |
| Donor age [ys] | median [range] | 59 [50-78] | 27 [18-35] | ≥50 | ≤35 | 60 [50-85] | 25 [18-35] |
| Diagnosis | AML | 63 | 60 | 0 | 0 | 100 | 100 |
|  | MDS/MPN | 37 | 40 | 100 | 100 | 0 | 0 |
| Disease risk | high/very high DRI or IPSS-R high/very high or ELN adverse risk [%] | 44 | 46 | 25 | 21 | 28 | 25 |
| Karnofsky Score | ≥90 [%] | 59 | 59 | 53 | 52 | 53 | 55 |
| Conditioning | MAC [%] | 17 | 14 | 34 | 29 | 47 | 39 |
| GVHD Prophylaxis | ATG [%] | 52 | 86 | 12 | 34 | 10 | 37 |
|  | PTCy [%] | 2 | 2 | not available | | not available | |
| Stem cell source | PBSC [%] | 97 | 98 | 97 | 90 | 96 | 89 |
| Sex match | patM-donF [%] | 30 | 9 | 31 | 12 | 26 | 11 |
| CMV match | pat-/don- [%]  pat+/don+ [%] | 23  43 | 35  41 | 21  37 | 32  26 | 19  42 | 24  26 |
| AML  5-yr EFS  5-yr OS  5-yr CIR  5-yr NRM | [%] (95%-CI) | 41 (36-45)  45 (40-50)  34 (30-39)  25 (22-29) | 42 (38-45)  48 (44-52)  34 (30-37)  25 (22-28) | -  -  -  - | -  -  -  - | 41 (38-43)  47 (45-49)  41 (39-43)  20 (17-24) | 44 (42-46)  47 (44-49)  35 (33-37)  17 (15-20) * |
| MDS/MPN  5-yr EFS  5-yr OS  5-yr CIR  5-yr NRM | [%] (95%-CI) | 41 (36-47)  47 (42-52)  30 (25-35)  29 (24-34) | 46 (42-51)  54 (50-59)  25 (22-29)  28 (25-32) | 25 (21-29)  34 (30-38)  50 (46-54)  26 (22-29) | 31 (27-34)  37 (34-41)  37 (34-40)  32 (29-35) | -  -  -  - | -  -  -  - |

Legend: N, number of donors; ys, years; HCT, hematopoietic cell transplantation; AML, acute myeloid leukemia; MDS/MPN, myelodysplastic/ myeloproliferative neoplasms; DRI, disease-risk index; ELN, European LeukemiaNet; MAC, myeloablative conditioning; GVHD, Graft-versus-host disease; ATG, anti-thymocyte globulin; PTCy, post-transplantation cyclophosphamide; PBSC, Peripheral Blood Stem Cells; CMV cytomegalovirus; pat, patient; don, donor; M, male; F, Female, -, negative; +, positive; MSD, matched sibling donor; mUD, matched unrelated donor; DRST, Deutsches Register für Stammzelltransplantation/German Registry for Stem Cell Transplantation; CIBMTR, Center for International Blood and Marrow Transplant Research; *NRM reported for period from 2016-2018; EFS, event-free survival; OS, overall survival; CIR, cumulative incidence of relapse; NRM, cumulative incidence of non-relapse mortality.

## Figure S1. Numbers of HLA-identical sibling and HLA-compatible unrelated donor transplants over time.


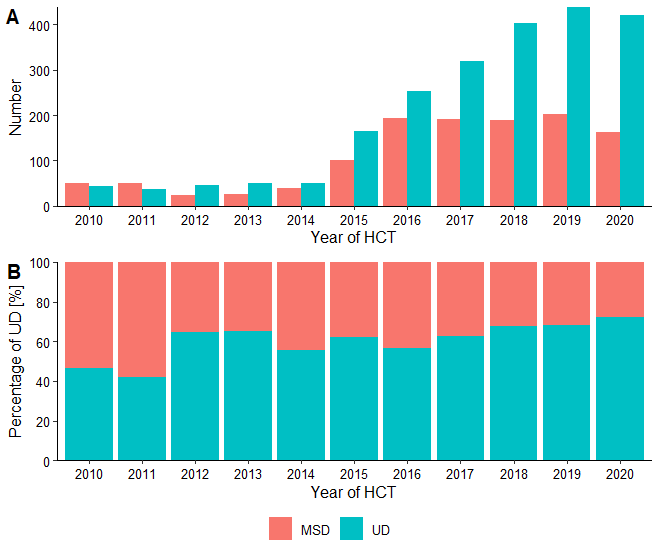


## Figure S2. Event-free and overall survival, and cumulative incidences of relapse, non-relapse mortality, acute GVHD grades II-IV, and chronic GVHD of any severity by donor type


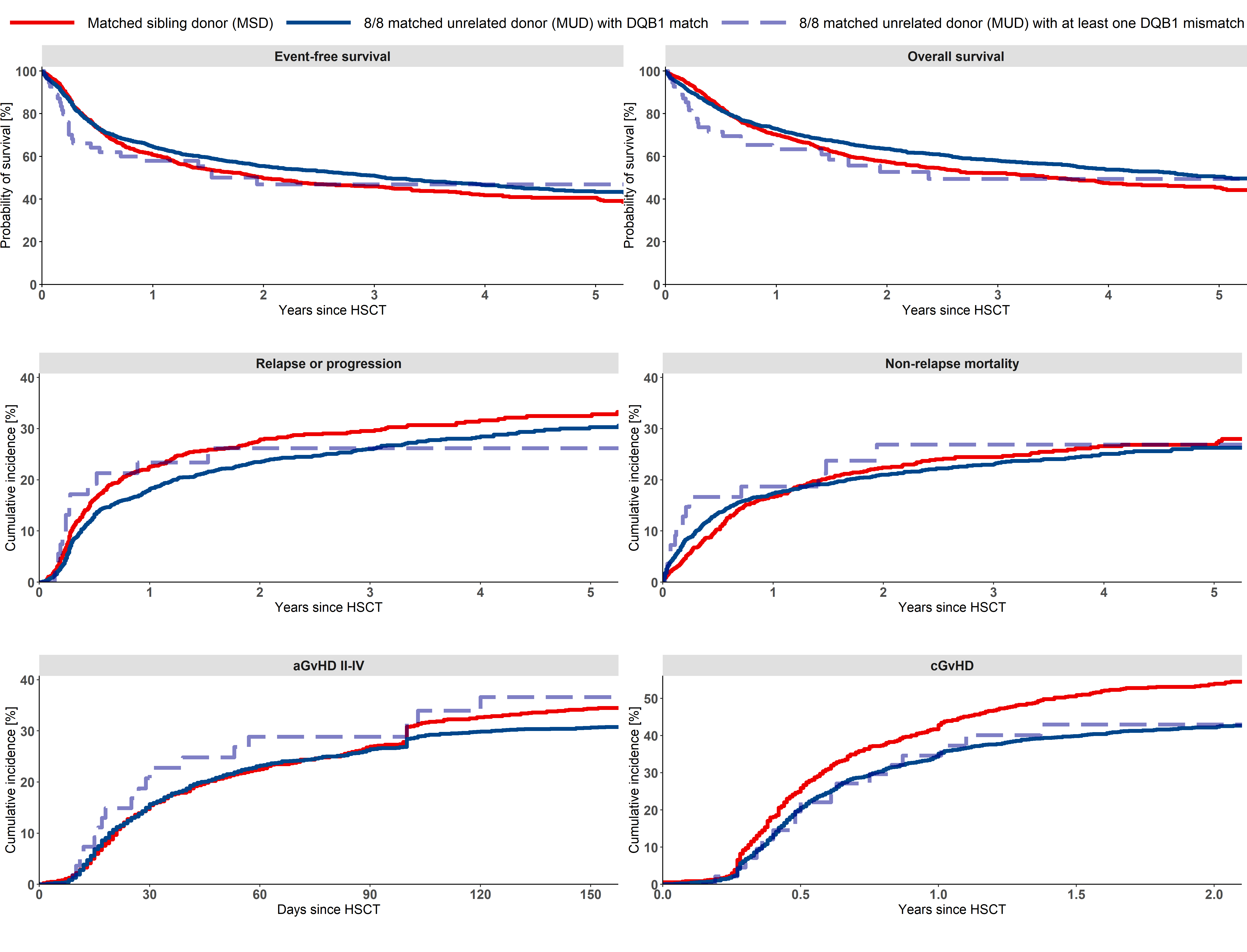

Supplement: Supplementary file 1 — Supplement [file 41375_2025_2724_MOESM1_ESM.docx]
